# A Postbiotic Derived from *Lactobacillaceae* Protects Intestinal Barrier Function in a Challenge Model Using Colon Organoid Tubules

**DOI:** 10.3390/foods14071173

**Published:** 2025-03-27

**Authors:** Colin I. Cercamondi, Igor Bendik, Erik Eckhardt, Tim Mak, Nicole Seifert, Karin Kuratli, Nathalie Richard, Balint Tamasi, Bernd Mussler, Eva Wintergerst

**Affiliations:** DSM-Firmenich AG, Wurmisweg 576, 4303 Kaiseraugst, Switzerland; igor.bendik@dsm-firmenich.com (I.B.); erik.eckhardt@dsm-firmenich.com (E.E.); tim.mak@trilliome.com (T.M.); nicole.seifert@dsm-firmenich.com (N.S.); karin.kuratli@dsm-firmenich.com (K.K.); nathalie.richard@dsm-firmenich.com (N.R.); balint.tamasi@dsm-firmenich.com (B.T.); bernd.mussler@dsm-firmenich.com (B.M.); e.wintergerst@bluewin.ch (E.W.)

**Keywords:** postbiotics, *Limosilactobaccilus fermentum*, *Lactobacillus delbrueckii*, organoid tubules, intestinal barrier, cytokines

## Abstract

Postbiotics may help strengthen intestinal barrier function. This study assessed the effects of a postbiotic derived from *Limosilactobacillus fermentum* and *Lactobacillus delbrueckii* subsp. *lactis* on epithelial barrier and cytokine production. Human-derived colon tubules were cultured on chips for 15 days. On day 8, the epithelial barrier was disrupted with 0.7 μM afatinib. Postbiotic doses of 5, 10, or 20 mg/mL were added on days 6, 8, 11, and 13. Trans-epithelial electrical resistance (TEER) was measured on days 6, 8, 11, 13, and 15, along with phase contrast imaging. Cytokine levels were measured on day 13. All three postbiotic concentrations resulted in better TEER recovery on day 15 vs. the control (*p* < 0.001). On day 13, 10 and 20 mg/mL increased TEER (*p* < 0.001), but only 20 mg/mL did on day 11 (*p* < 0.05). Phase imaging confirmed the dose-dependent effect. The 20 mg/mL dose more effectively reduced CCL2, CX3CL1, CXCL1, CXCL5, IL-8, IL-11, and IL-4 than the other doses (*p* < 0.01), and 10 mg/mL more effectively reduced CCL2, CXCL1, CXCL10, IL-10, IL-11, and IL-23 than 5 mg/mL (*p* < 0.01). In a colonic organoid model, the *Lactobacillaceae*-derived postbiotic prevented drug-induced epithelial damage, enhanced recovery, and modulated cytokine secretion towards a more anti-inflammatory profile in a dose-dependent manner.

## 1. Introduction

Barrier function is a crucial aspect of gut health, preventing harmful substances from entering the bloodstream and ensuring efficient nutrient absorption [1]. Current knowledge suggests that biotic interventions can support and enhance the function of epithelial cells in the gut barrier [2,3,4]. Postbiotics are emerging biotics and are defined as a preparation of inanimate microorganisms and/or their components that confer a health benefit to the host [5]. They may directly strengthen barrier function by enhancing tight junction protein expression [6] or indirectly by their anti-inflammatory, antimicrobial, and microbiota-shaping properties [6,7,8,9,10]. Components of postbiotic preparations that may be responsible for these effects include, for example, exopolysaccharides, which have been shown to upregulate tight junction proteins in the gut and possess antioxidant properties that help to reduce oxidative stress and inflammation [6,7]. Moreover, short-chain fatty acids or bacteriocins produced by the bacteria used in postbiotic preparations may enhance barrier function by protecting against intestinal inflammation or through their antimicrobial properties, respectively [7]. In addition, postbiotics have been reported to prevent common infectious diseases in children, likely by stimulating the innate and acquired immune system [11,12]. In contrast to probiotics, postbiotics do not face viability concerns and offer enhanced safety and stability. This increased stability is associated with a longer shelf life, as well as easier transport, storage, and handling [7,13,14].

Clinical evidence for the benefits of different postbiotics is available [15,16,17], and the effector molecules involved along with the potential mechanisms for the beneficial effects have been assessed in in vitro and in vivo models [14,18]. However, the commonly used in vitro and in vivo models have limitations and may not effectively replicate the human gastrointestinal environment [19]. For example, in vitro models using a monolayer of immortalized intestinal cell lines derived from tumors (e.g., Caco-2 and HT-29 cells) do not differentiate into normal gastrointestinal tissue [20] and cannot fully reproduce the complex gastrointestinal environment, which includes various types of epithelial cells [19,21,22]. Animal models have species-specific cell structure and composition in their gastrointestinal environment, as well as microbial communities, that are different to those of humans [23]. In addition, they are more complex to manage and more expensive than in vitro models and come with ethical concerns [19]. As a result, more suitable alternatives called organoids, which better mimic the intestinal structure, were developed about a decade ago [24,25].

Intestinal organoids are self-organized three-dimensional tissue cultures deriving from stem cells, which better simulate the in vivo conditions by developing into a polarized monolayer, which, unlike cell lines, is composed of non-transformed, primary cells [19]. They express various stem cell-derived cell types, including tuft cells and M cells, as well as cells that were previously difficult to culture, such as enteroendocrine cells [26]. Organoids preserve many of the advantages of existing in vitro models (e.g., high throughput) while moving the whole model substantially closer to the real-life situation. They are expected to become the new standard for in vitro testing, aiding in better preclinical decision-making, reducing the need for animal testing, and uncovering the mechanisms of action. The purpose of this study was, therefore, to further elucidate the potential mechanisms of action behind previously reported benefits [16,17,27,28,29] of a specific postbiotic derived from the co-fermentation of *Limosilactobacillus (L.) fermentum* and *Lactobacillus (L.) delbrueckii* subsp. *lactis* (historically referred to as “Lactobacillus LB” and once believed to be a single strain of *L. acidophilus*). This was achieved by testing this specific postbiotic for the first time in a human-derived colon organoid model using novel chip technology and assessing its effects on epithelial barrier function (including cell morphology) and cytokine production.

## 2. Materials and Methods

**Figure 1:**
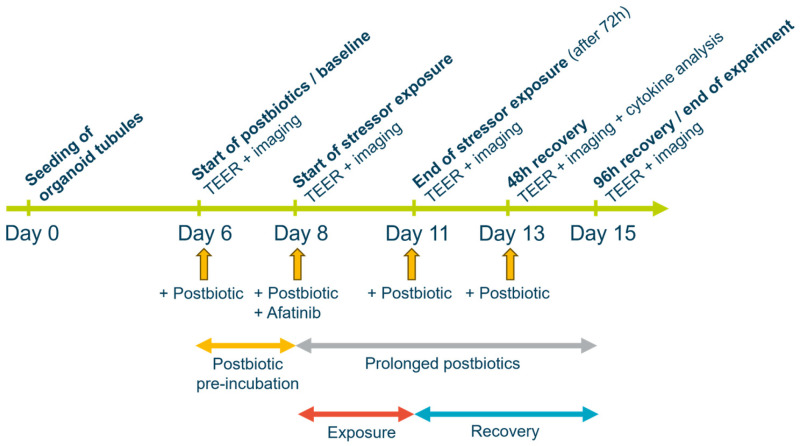
Experimental set up. TEER (Trans-Epithelial Electrical Resistance).

Cell culture: OrganoReady^®^ Colon Organoid 3-lane 64 (MI-OR-CU, MIMETAS, Oegstgeest, The Netherlands) plates were used to study the effects of the postbiotics on the epithelial barrier. The plates consisted of 64 individual microfluidics chips that were seeded with intestinal organoid tubules on day 0 (Figure 1). Organoid tubules were established from an organoid line derived from a healthy colon biopsy of a single human donor. Each chip constituted of three channels, a middle channel filled with an extracellular matrix that provided support for the developed organoid tubule in the right channel and the opposite channel containing the cell culture media. The culture media were added to the inlets and outlets of the outer channels, and perfusion was initiated upon placement of the plates onto the OrganoFlow^®^ (MI-OFPR-L, MIMETAS, Oegstgeest, The Netherlands), which was set at a 14° inclination and 8 min interval while rocking inside an incubator (37 °C, 5% CO_2_). Media were refreshed every 2–3 days.

Experimental set up and exposure: A total of eight different experimental arms were tested: control (not challenged; n = 10), challenged control (n = 10), three different postbiotic concentrations not challenged (n = 5), and three different postbiotic concentrations with challenge (n = 5 or n = 6). The postbiotic preparation was heat-inactivated *L. fermentum* and *L. delbrueckii* subsp. *lactis* as well as components of the fermented culture medium, including peptides, amino acids, carbohydrates, and minerals (Humiome^®^ Post LB, DSM-Firmenich, Houdan, France). Standard quality assessments of this postbiotic preparation included confirming the presence of at least 60 × 10^9^ structurally intact, inanimate bacterial bodies per gram, as determined by flow cytometry. Furthermore, the bioactivity of the postbiotic was assessed by testing its in vitro inhibitory activity on the invasion of Caco-2 epithelial cells by *Salmonella* spp. with an established threshold of ≥90% inhibition [30]. The postbiotic was added to the apical side of the organoid tubules on day 6 of culture (baseline; Figure 1). Three different postbiotic concentrations were tested (5, 10, and 20 mg/mL), corresponding to ~0.3 to 1.2 × 10^9^ inanimate cells and reflecting effective dosages tested in previous studies [27,29,30]. The standard culture media condition was used as a control. For the experimental arms with challenge, forty-eight hours later, on day 8 of culture, the tyrosine kinase inhibitor afatinib, which is known to disrupt the epithelial barrier [31,32], was added at 0.7 μM to the apical side of the organoid tubules. Simultaneously, the postbiotic preparation was re-dosed. On day 11 from culture, after 72 h of afatinib exposure, the media were refreshed, and the postbiotic preparation was re-dosed. On day 13 from culture (48 h recovery), the postbiotic preparation was re-dosed, and the organoid tubules were kept in culture until day 15 (96 h recovery/end of experiment).

Assessment of the epithelial barrier function: Phase contrast imaging was performed to assess cells’ morphology on days 6, 8, 11, 13, and 15 (Figure 1). Organoid tubules were imaged on the ImageXpress Micro 4 system (Molecular Devices LLC, San Jose, CA, USA) at 4× magnification. Epithelial barrier function was assessed by measuring trans-epithelial electrical resistance (TEER) with an automated multichannel impedance spectrometer (OrganoTEER^®^, MIMETAS, Oegstgeest, The Netherlands) on days 6, 8, 11, 13, and 15. An electrode board that matched the 3-lane 64 OrganoReady^®^ plate format was sterilized with 70% ethanol an hour prior to the measurement. The OrganoReady^®^ Colon Organoid 3-lane 64 plate was placed on a flat surface inside the laminar flow cabinet to equilibrate to room temperature 30 min before the measurement. Subsequently, the OrganoReady^®^ plate was placed in the OrganoTEER^®^ device, and a single timepoint impedance measurement was performed with a frequency range of 10 Hz to 1 MHz (41 points; precision, 0.2). TEER values were reported per chip in ohms (Ω) and were corrected for the surface area of the tubule–ECM interface (0.0057 cm^2^).

Cytokine assessment: Cytokines in media supernatants collected on day 13 in the challenged arms, were quantified using Luminex technology (LiquiChip Workstation IS 200, Qiagen, Hilden, Germany) and Luminex Screening Assay kits (R&D Systems, Inc., Minneapolis, MN, USA), according to the manufacturer’s instructions. The data were acquired with the Luminex IS 2.3 software and evaluated with the LiquiChip Analyser 1.0 software (Qiagen, Hilden, Germany). The following cytokines were assessed: CCL2/MCP-1, CCL4/MIP-1β, CX3CL1/Fractalkine, CXCL1/GROα, CXCL10/IP-10, CXCL5/ENA-78, IL-8/CXCL8, FGF basic/FGF2, GM-CSF, IFN-γ, IL-1β, IL-2, IL-4, IL-6, IL-10, IL-11, IL-13, IL-17, IL-23, and TNF-α.

Data analysis: TEER and cytokine data are presented as means and standard errors of the means (mean ± SEM). To analyze the longitudinal TEER responses between certain experimental arms, we estimated a marginal linear model with generalized least squares (GLS) using the R package *nlme* (R version 4.4.2). The model assumes an unstructured correlation matrix across measurements within the same replicate and separate error variances over time. We adjusted for baseline differences in TEER values by including them as covariates in the regression model. Group comparisons were performed by defining the appropriate contrasts and adjusting for multiple testing with the R package *multcomp* (R version 4.4.2). The results from this model are presented as point estimates of mean differences between groups alongside the corresponding 95% confidence intervals and *p*-values for the specific group comparisons. *p*-values < 0.05 were considered as statistically significant.

The cytokine concentrations were compared between the challenged control and the three challenged different postbiotic concentrations using the exact Wilcoxon rank-sum test. To control the false discovery rate across various cytokine measurements and postbiotic dosages, the *p*-values were adjusted with the Benjamani–Hochberg procedure. *p*-values < 0.05 were considered as statistically significant.

## 3. Results

### 3.1. Induction of Epithelial Barrier Damage

**Figure 2:**
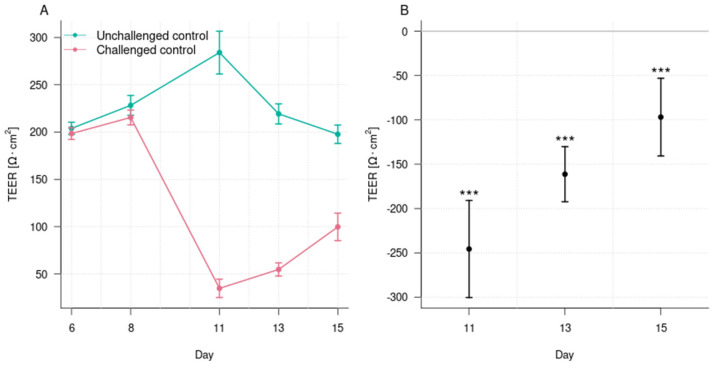
Trans-epithelial electrical resistance (TEER) for the unchallenged control and the challenged control on days 6, 8, 11, 13, and 15 (panel (**A**)). For the challenged control, 0.7 μM afatinib was added on the apical side on day 8 and kept in the media until day 11. Data are shown as means with ± 1 standard errors as whiskers. Mean difference estimates of TEER, with their 95% multiplicity-adjusted confidence intervals, for the challenged control compared to the unchallenged control on days 11, 13, and 15 (panel (**B**)). n = 10. Asterisks indicate a significant difference: *** *p* < 0.001. All *p*-values were adjusted for multiple testing.

**Figure 3:**
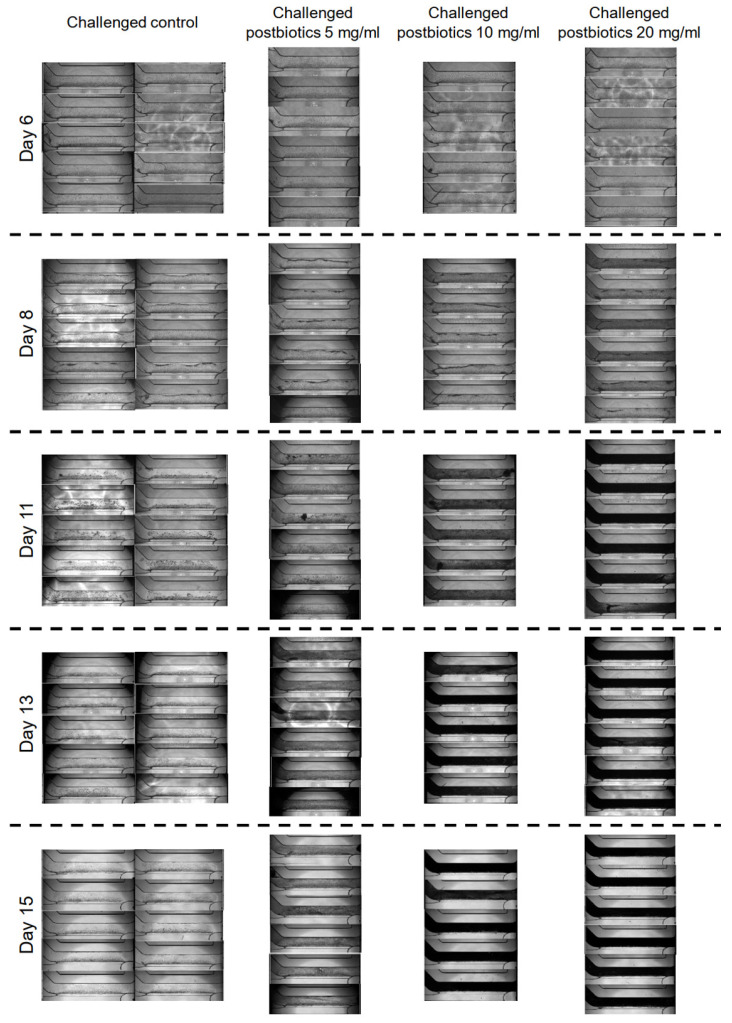
Phase contrast imaging of the organoid tubules for the challenged control and the three challenged postbiotic concentrations. n = 10 for the challenged control, n = 6 for 5 and 20 mg/mL postbiotics, n = 5 for 10 mg/mL postbiotics.

The exposure of the organoid tubules to afatinib for 72 h resulted in a significant reduction in TEER as compared with the unchallenged control at day 11 (35 ± 10 Ω × cm^2^ vs. 284 ± 23 Ω × cm^2^; *p* < 0.001) (Figure 2). The TEER for the challenged control remained lower than the unchallenged control at day 13 (55 ± 7 Ω × cm^2^ vs. 219 ± 11 Ω × cm^2^; *p* < 0.001) and day 15 (100 ± 15 Ω × cm^2^ vs. 198 ± 10 Ω × cm^2^; *p* < 0.001). These results demonstrate that 0.7 μM afatinib was able to disrupt the epithelial barrier function and that recovery did not happen until the end of the experiment. The disruption of the epithelial barrier was also confirmed in the phase imaging where a disruption of the cell layer and a reduction in the epithelial cell number were observed (Figure 3). Thus, it is suitable as a model to assess the effects of postbiotics in preserving and recovering the barrier function.

### 3.2. Dose-Dependent Effect of the Postbiotic in Preventing Epithelial Barrier Damage

**Figure 4:**
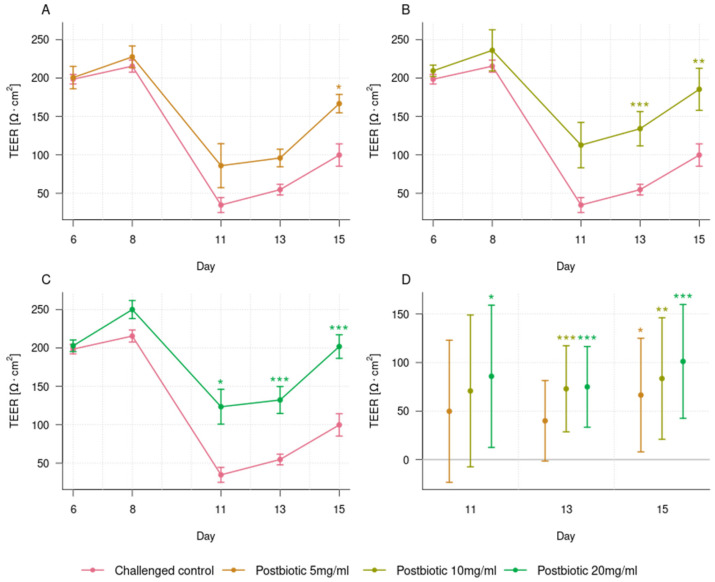
Trans-epithelial electrical resistance (TEER) for the three challenged postbiotic concentrations and the challenged control before the challenge with afatinib on days 6 and 8, after the challenge on day 11, and during recovery on days 13 and 15 (panels (**A**–**C**)). Data are shown as means with ± 1 standard errors as whiskers. Mean difference estimates of TEER, with their 95% multiplicity-adjusted confidence intervals, for the three concentrations compared to the challenged control on days 11, 13, and 15 (panel (**D**)). n = 10 for the challenged control, n = 6 for 5 and 20 mg/mL postbiotics, and n = 5 for 10 mg/mL postbiotics. Asterisks indicate significant differences in the recovery period (day 11 to 15): * *p* < 0.05, ** *p* < 0.01, and *** *p* < 0.001. All *p*-values were adjusted for multiple testing.

The incubation of the organoid tubules with 5 mg/mL postbiotics did not result in a statistically significant protection of the epithelial barrier from the disruption caused by afatinib, as assessed by TEER at day 11 (35 ± 10 Ω × cm^2^ for the challenged control vs. 86 ± 29 Ω × cm^2^ for the challenged 5 mg/mL postbiotics; *p* = 0.376; Figure 4A,D). Nevertheless, the phase imaging clearly shows that more cells remain present and intact in the challenged organoid tubules with the postbiotics compared to the challenged control (Figure 3). Furthermore, on day 13, the continuous incubation of 5 mg/mL postbiotics to the organoids challenged with afatinib showed a trend for better recovery of the epithelial barrier function compared with the challenged control (55 ± 7 Ω × cm^2^ vs. 96 ± 11 Ω × cm^2^; *p* = 0.066; Figure 4A,D). On day 15, continuous incubation with the postbiotics resulted in better recovery (100 ± 15 Ω × cm^2^ vs. 167 ± 12 Ω × cm^2^; *p* < 0.05; Figure 4A,D). These differences in barrier function were also noticeable in the phase imaging where more cells were found in the challenged organoid tubules with postbiotics when compared to the challenged control on days 13 and 15 (Figure 3).

On day 11, the incubation of the organoid tubules with 10 mg/mL postbiotics provided similar results as the 5 mg/mL postbiotics with no statistically significant protection of the epithelial barrier from the disruption caused by afatinib, as assessed by TEER (35 ± 10 Ω × cm^2^ for the challenged control vs. 113 ± 30 Ω × cm^2^ for the challenged 10 mg/mL postbiotics; *p* = 0.101; Figure 4B,D). However, the phase imaging clearly showed that more cells remained present and intact in the challenged organoid tubules with postbiotics than in the challenged control (Figure 3). In contrast to 5 mg/mL postbiotics, continuous incubation of 10 mg/mL postbiotics resulted in higher TEER values at day 13 compared to the challenged control (55 ± 7 Ω × cm^2^ vs. 134 ± 22 Ω × cm^2^; *p* < 0.001; Figure 4B,D), and the TEER values remained higher at day 15 (100 ± 15 Ω × cm^2^ vs. 185 ± 27 Ω × cm^2^; *p* < 0.001; Figure 4B,D). This difference in intestinal barrier integrity was also observable in the phase imaging where more cells were visible in the chips challenged with the postbiotics when compared to the challenged control on days 13 and 15 (Figure 3).

For the organoid tubules incubated with 20 mg/mL postbiotics, a positive effect on TEER was already seen on day 11 (35 ± 10 Ω × cm^2^ for the challenged control vs. 123 ± 23 Ω × cm^2^ for the challenged organoid tubules with postbiotics; *p* < 0.05; Figure 4C,D), indicating a stronger protective effect of the 20 mg/mL than the two lower concentrations. On day 13 (55 ± 7 Ω × cm^2^ vs. 132 ± 18 Ω × cm^2^; *p* < 0.001) and day 15 (100 ± 15 Ω × cm^2^ vs. 202 ± 15 Ω × cm^2^; *p* < 0.001), the epithelial barrier function was better for the organoid tubules challenged with 20 mg/mL postbiotics compared to the challenged control (Figure 4C,D). Phase imaging also indicated a preservation of the epithelial layer’s integrity, even though accurate interpretation of the images was difficult due to the high concentration of the postbiotics, leading to black staining (Figure 3).

### 3.3. Effect of the Postbiotic on Cytokine Release Profile

The quantification of cytokines in the epithelial layer supernatant on day 13 demonstrated that the addition of 20 mg/mL postbiotics significantly reduced the secretion of CCL2, CX3CL1, CXCL1, CXCL5, IL-4, IL-8, IL-10, IL-11, and IL-23 in comparison with the afatinib-challenged control. For CXCL10, a reduction trend was observed (Table 1). A significant reduction in certain cytokines was also observed with the 5 mg/mL and 10 mg/mL concentrations of the postbiotics. With 10 mg/mL postbiotics, a very similar pattern to that observed with 20 mg/mL was noted, with the same cytokines being significantly reduced when compared with the afatinib-challenged control, except for CXCL5. With the 5 mg/mL postbiotics, only CCL2, CX3CL1, and CXCL1 were significantly reduced compared with the afatinib-challenged control. For IL-11, a trend was observed (Table 1). A comparison between the different postbiotic concentrations demonstrated a dose effect, with the 20 mg/mL being more effective than both the 10 mg/mL and the 5 mg/l in reducing the secretion of CCL2, CX3CL1, CXCL1, CXCL5, IL-8, IL-11, and IL-4 (*p* values for 20 mg/mL vs. 10 mg/mL and 20 mg/mL vs. 5 mg/mL: *p* = 0.009). In addition, the 10 mg/mL postbiotic dose was also more effective than the 5 mg/mL in reducing the concentrations of CCL2, CXCL1, CXCL10, IL-10, IL-11, and IL-23 (*p* = 0.009). For the following cytokines, values were below the detection limit or borderline to the detection limit: CCL4/MIP-1β, IFN-γ, IL-1β, IL-2, IL-6, IL-13, IL-17, FGF basic/FGF2, GM-CSF, and TNF-α.

## 4. Discussion

Our in vitro study, testing a specific postbiotic derived from *L. fermentum* and *L. delbrueckii* subsp. *lactis* for the first time in organoids, demonstrated a dose-dependent beneficial effect. It preserved the intestinal barrier function from acute damage and restored the epithelial barrier following disruption. These effects were observed when the postbiotic was added to the organoid tubules 48 h before the challenge with afatinib and when continuously re-dosed at three different timepoints (days 8, 11, and 13), simulating a prevention approach where a beneficial compound is continuously provided to mitigate the negative impact of sudden challenges. We demonstrated that on day 11, 20 mg/mL postbiotics better protected the organoids from the drug-induced TEER decrease than 5 or 10 mg/mL. Furthermore, we showed that on day 13 or 15, TEER recovered to higher values with the use of higher postbiotic concentrations. This dose-dependent effect was also noticeable in the phase contrast images, with more intact cells being present in the organoid tubules incubated with higher postbiotic concentrations. Furthermore, this study indicates that our postbiotic possesses anti-inflammatory properties, and its protective effects on the intestinal barrier seem closely linked to these properties as we also observed dose-dependent effects of the postbiotic on cytokine secretion, with 20 mg/mL being generally more effective in inhibiting the release of these molecules compared to lower doses.

Lactobacillus species (including those reclassified into other genera in 2020) are the most widely used and studied probiotics [33]. Our results align with previous studies demonstrating that the same postbiotic preserves tight junctions and epithelial integrity in models with conventional cells [29,34,35] and in vivo [36]. Note that in the past, it was mistakenly believed that the postbiotic is derived from a single strain of *L. acidophilus*, commercially known as “Lactobacillus LB”. Studies with different strains of *L. delbrueckii* subsp. *bulgaricus* have also shown beneficial effects on the intestinal epithelium [37]. Similarly, our results correspond with the few existing studies that have tested other Lactobacillus-derived postbiotics in organoids. These studies reported enhanced stem cell proliferation, reduced inflammation, and improved tight junctions when postbiotics were used in challenge models [38,39,40,41]. In contrast to the previous studies with organoids, our model allowed for TEER assessments, a direct measurement of intestinal barrier function. Organoid structures include various intestinal cell types, thereby better simulating the complex intestinal environment than conventional cell line models. In recent years, these “mini-intestine” organoids have risen as valuable alternatives to traditional in vitro models for assessing the effects of biotics [19,42,43]. Considering that organoids are more cost-efficient than animal models and superior to traditional in vitro models in simulating a human-like intestinal environment, due to their inclusion of several distinct cell types and better reflection of key structural properties, they could be the model of choice for high-throughput preclinical screening of biotics. Indeed, in our study, we established an effective model for challenging intestinal integrity and assessing the effects of the postbiotic on the cytokine profile. This provided insights into potential mechanisms of action and demonstrated that organoid tubules are a valuable tool for investigating the interaction of postbiotics with intestinal tissue, their impact on intestinal barrier function, and their immunomodulatory effects. In addition, our study can act as a basis for developing models where intestinal integrity is challenged in a different way and where organoid tubules maybe co-cultured with bacteria, such as a model including commensal and pathogenic bacteria. Thus, further studies using organoid tubules with added complexity, such as incorporating microbiota and including transcriptomics for a more comprehensive analysis, would be interesting to further investigate aspects of host–postbiotic interactions, such as the impact on cell gene expression and microbiota effects including the neutralization of pathogens.

Our study focuses on the disruption of the intestinal barrier, which is found in diarrhea; various gastrointestinal diseases (e.g., inflammatory bowel disease, celiac disease, colon carcinoma, etc.) and also extra-intestinal disorders (e.g., chronic liver disease, type 1 diabetes, obesity, etc.) [44]. Although, diarrhea could have various etiologies and result from various alterations in intestinal mechanisms, the reduced effectiveness of the epithelial barrier is one of the potential contributing factors [45,46], and clinical studies have demonstrated that postbiotics help reduce diarrhea and its symptoms. In clinical studies, the postbiotic tested in our study, helped reduce diarrhea and associated pain and bloating in adult patients with irritable bowel syndrome with diarrhea [16], and it also helped reduce chronic diarrhea of various origins in both adults [17] and children [27,28,29]. Furthermore, a postbiotic based on *L. paracasei* was associated with reduced acute gastroenteritis [11,12]. A postbiotic based on more than one species like ours contains a broader spectrum of bioactive compounds than a single-species postbiotic and has, therefore, likely an enhanced and/or wider range of physiological effects. These compounds interact with the host’s epithelial cells via their toll-like or G-coupled receptors [47], and this can regulate various physiological processes, such as the secretion of cytokines. Indeed, our study demonstrated that the postbiotic influenced the secretion of various cytokines (CCL2, CXCL1, CXCL5, CXCL10, CX3CL1, IL-4, IL-8, IL-10, IL-11, and IL-23), with several showing dose-dependent effects, supporting the hypothesis that our postbiotic possesses immunomodulatory properties. Not many studies so far have assessed the effects of postbiotics on cytokine profiles. A study using the same postbiotic indicated some immunomodulatory effects based on changes in IL-17f and IL-12α using a murine challenge model [48]. Other studies demonstrated a reduction in CCL2 using *L. paracasei*-derived postbiotics in an in vitro inflammatory bowel disease model [49] or found that cell-free supernatants from different species (e.g., *L. acidophilus*) downregulated IL-8 expression in HT-29 cells [50]. Since inflammation and inflammatory diseases have been linked to the disruption of intestinal barrier integrity [1,51,52], downregulating the secretion of inflammation-related molecules likely indicates the mechanism by which the tested postbiotic helps preserve and restore intestinal integrity.

One of the strengths of our study is its novelty in using organoids for the first time to test this specific postbiotic, providing stronger evidence by employing a model that is closer to real-life conditions than previous studies. We also provide novel insights into the potential mechanism of the postbiotic’s action by demonstrating its effect on the secretion of inflammatory factors and identifying a dose-dependency of this effect. Our study also has limitations, including the assessment of cytokine concentrations at only one timepoint, which may not reflect peak cytokine secretion, and the inability to normalize these concentrations (e.g., based on total protein content) due to limited sample materials. For the same reason, we did not include any transcriptomic analysis. The assessment of phase contrast images was challenging due to obstructions in the view, which were likely caused by the accumulation of heat-inactivated bacteria fragments in the media when higher postbiotic concentrations were repeatedly added. Consequently, it was difficult to correlate the protective effect observed on TEER with any morphological characteristics of the epithelial barrier at higher postbiotic concentrations. The fact that the highest non-challenged postbiotic concentration did not develop a higher TEER than the non-challenged control, even with repeated postbiotic administration over time, ensures that the postbiotics themselves did not form a biofilm that interfered with the TEER measurement (the TEER results and phase imaging of the three different postbiotic concentrations not challenged in comparison with the unchallenged control are available in Supplementary Figures S1 and S2).

## 5. Conclusions

Our organoid study has demonstrated the effectiveness of a postbiotic resulting from the co-fermentation of *L. fermentum* and *L. delbrueckii* in preserving and restoring the intestinal barrier function from acute damage in a dose-dependent manner. Additionally, this study offers insights into the mechanism of the postbiotic’s protective action by demonstrating its immunomodulatory effects, evidenced by the reduced secretion of certain cytokines. These findings help to explain previously reported benefits of this postbiotic and substantiate its potential as a nutritional intervention to enhance gut health. Further in vitro and in vivo studies are required to identify the specific postbiotic compounds responsible for these effects and to validate these findings in clinical settings.

## Figures and Tables

**Table 1:** Cytokine concentrations on day 13 in the three challenged postbiotic concentrations and the challenged control.

Variable [pg/mL]	Challenged Control	Challenged Postbiotics 5 mg/mL	*p*-Value	Challenged Postbiotics 10 mg/mL	*p*-Value	Challenged Postbiotics 20 mg/mL	*p*-Value
CCL2	3450 ± 299	2010 ± 144	0.013	1018 ± 97	0.013	101 ± 20	0.009
CX3CL1	1186 ± 63	591 ± 49	0.009	489 ± 38	0.013	164 ± 17	0.009
CXCL1	5828 ± 237	4443 ± 153	0.009	2596 ± 141	0.013	564 ± 66	0.009
CXCL10	62 ± 19	35 ± 10	0.290	7 ± 0.3	0.013	24 ± 8	0.074
CXCL5	122 ± 6	125 ± 4	1.000	125 ± 6	0.837	52 ± 10	0.009
IL-8	2238 ± 282	3224 ± 751	0.459	1630 ± 193	0.197	395 ± 65	0.009
IL-10	19 ± 4	11 ± 2	0.216	3 ± 0.3	0.013	8 ± 2	0.032
IL-11	5840 ± 330	4553 ± 315	0.051	2598 ± 227	0.013	290 ± 52	0.009
IL-23	784 ± 151	519 ± 71	0.216	172 ± 10	0.013	277 ± 87	0.017
IL-4	25 ± 2	20 ± 2	0.185	14 ± 1	0.013	3 ± 1	0.009

Mean ± SEM; n = 10 for the challenged control; n = 6 for 5 and 20 mg/mL postbiotics, n = 5 for 10 mg/mL postbiotics. *p*-values are derived from Wilcoxon rank-sum tests for the comparison of distribution locations of the three challenged postbiotic concentrations compared to the challenged control. To control the false discovery rate, *p*-values are adjusted with the Benjamani–Hochberg procedure. *p*-values < 0.05 were considered as statistically significant.

## Data Availability

The original contributions presented in this study are included in the article/Supplementary Material. Further inquiries can be directed to the corresponding author.

## Supplementary Materials

The following supporting information can be downloaded at https://www.mdpi.com/article/10.3390/foods14071173/s1, Figure S1. Trans-epithelial electrical resistance (TEER) for the three unchallenged postbiotic concentrations and the unchallenged control on days 6, 8, 11, 13, and 15; Figure S2. Phase contrast imaging of the organoid tubules for the unchallenged control and the three unchallenged postbiotics concentrations.
