# Supplementary material for: A Postbiotic Derived from Lactobacillaceae Protects Intestinal Barrier Function in a Challenge Model Using Colon Organoid Tubules
Source: Foods. 2025 Mar 27;14(7):1173. doi: 10.3390/foods14071173 (PMC11988720; doi:10.3390/foods14071173)
Supplement: Supplementary file 1 [file foods-14-01173-s001.zip › foods-3511179-supplementary.pdf]

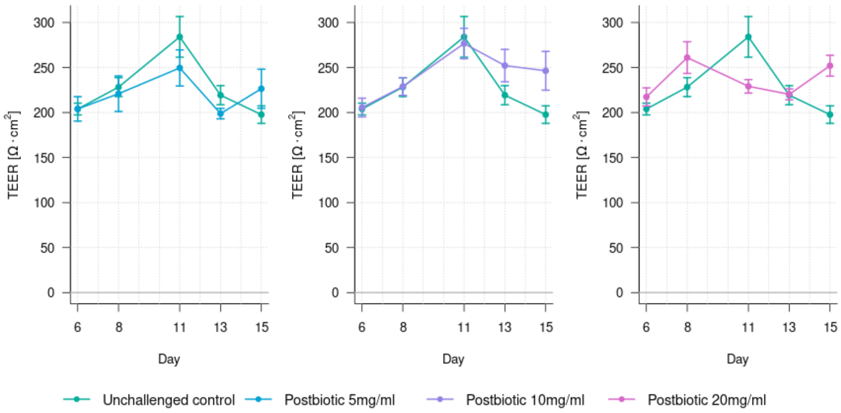

Figure S1. Trans-epithelial electrical resistance (TEER) for the three unchallenged postbiotic concentrations and the unchallenged control on days 6, 8, 11, 13 and 15. Data are shown as means with ± 1 standard errors as whiskers; n=10 for the unchallenged control; n=5 for the unchallenged postbiotics concentrations.

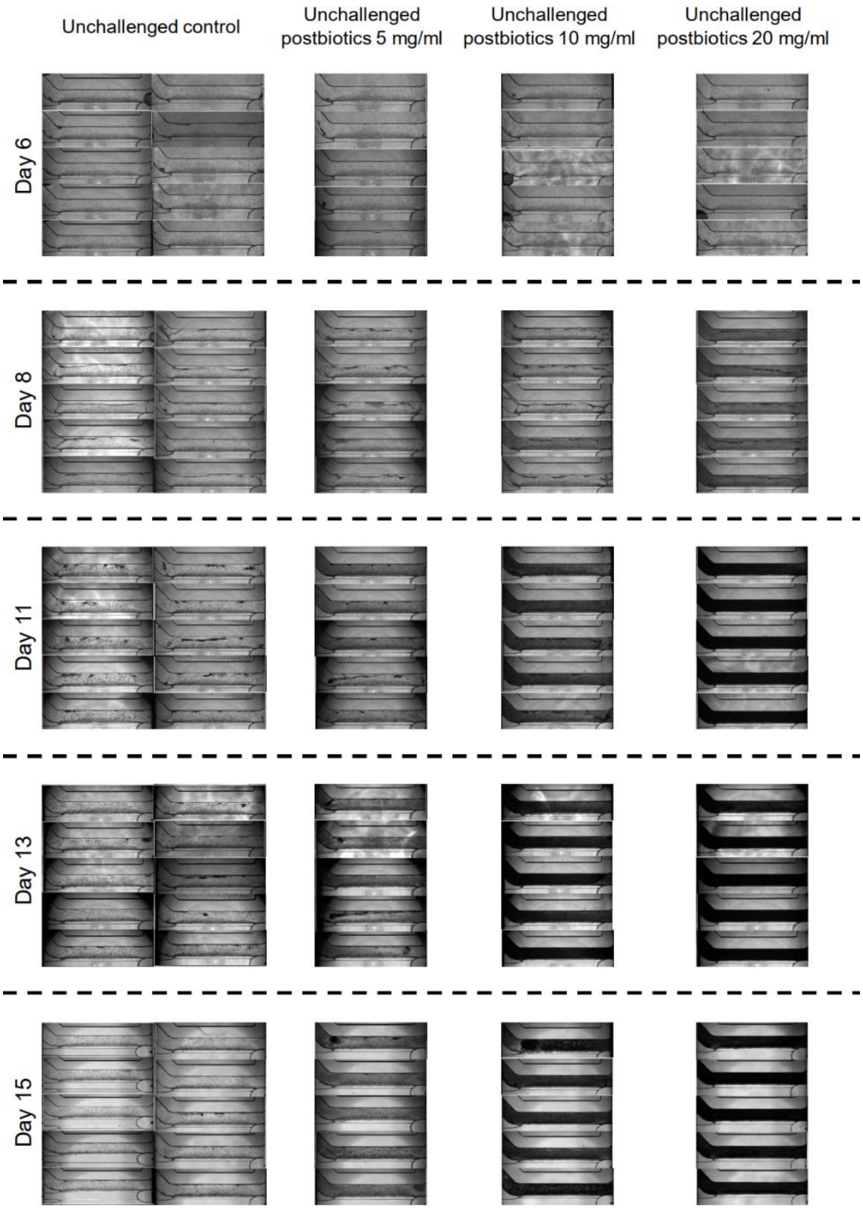

Figure S2. Phase contrast imaging of the organoid tubules for the unchallenged control and the three unchallenged postbiotics concentrations. n=10 for the unchallenged control; n=5 for the unchallenged postbiotics concentrations.
